# Correlation of factor XIII subunit A with factor XIII activity in a population of parturient women

**DOI:** 10.1007/s00404-024-07799-2

**Published:** 2024-11-04

**Authors:** M. L. Frevert, J. Bürgi, R. Brun, T. Hothorn, M. Rösslein, N. Ochsenbein-Kölble, C. Haslinger, W. Korte

**Affiliations:** 1 Department of Obstetrics, University Hospital Zurich, Frauenklinikstrasse 10, CH-8091 Zurich, Switzerland; 2 University of Zurich, Zurich, Switzerland; 3 Center for Laboratory Medicine, Hemostasis and Hemophilia Center, St. Gallen, Switzerland; 4 Epidemiology, Biostatistics and Prevention, Institute, University of Zurich, Zurich, Switzerland; 5 Swiss Federal Laboratories for Materials Research and Testing, Laboratory for Particles—Biology Interactions, EMPA, Lerchenfeldstrasse 5, CH-9014 St. Gallen, Switzerland

**Keywords:** Postpartum hemorrhage, PPH, Postpartum blood loss, Factor XIII activity, Factor XIII antigen

## Abstract

**Background:**

The role of factor XIII in acute bleeding situations is gaining more and more importance. It has previously been shown that prepartum factor XIII activity has a significant impact on postpartum blood loss. Whether factor XIII antigen behaves in a similar manner is unknown. As postpartum hemorrhage is one of the leading causes of maternal morbidity and mortality worldwide and factor XIII antigen determination might be available more readily in some centers as compared to factor XIII activity, this is an important question to answer, especially in the emergency situation of a postpartum hemorrhage.

**Objective:**

To assess the correlation of prepartum factor XIII antigen with prepartum factor XIII activity and to evaluate the correlation between prepartum factor XIII antigen on measured postpartum blood loss.

**Methods:**

This is a secondary analysis of a prospective cohort study which analyzed the impact of prepartum blood coagulation factor XIII activity on postpartum blood loss in 1300 women at the University Hospital Zurich, Switzerland between October 2015 and November 2016 (“*PPH-1300 study*”). Blood loss was quantified using a previously validated technique. The association of factor XIII activity and factor XIII antigen was assessed by means of a Spearman rank correlation and differences were displayed using Bland–Altman plot and Passing–Bablok regression. The effect of the prepartum factor XIII antigen on blood loss was estimated by continuous outcome logistic regression.

**Results:**

Prepartum factor XIII activity significantly correlated with prepartum factor XIII antigen (Spearman rank correlation coefficient for prepartum values 0.89, *p* < 0.001 and postpartum values 0.902, *p* < 0.001). Elevated values of prepartum factor XIII antigen showed a trend toward lower measured postpartum blood loss.

**Conclusion:**

The correlation of factor XIII activity with factor XIII antigen (subunit A) in a large real-world sample as well as an association of prepartum factor XIII antigen and postpartum blood loss is observed. Factor XIII antigen determination, a highly automatable test, could be useful in emergency situations such as a PPH (as well as other bleeding situations) if the determination of factor XIII activity is not possible. To evaluate whether FXIII replenishment reduces blood loss is the focus of ongoing studies.

**Supplementary Information:**

The online version contains supplementary material available at 10.1007/s00404-024-07799-2.

## Introduction

Postpartum hemorrhage (PPH), defined as a blood loss of 500 ml or more within 24 h after birth, is a major cause of maternal mortality and morbidity worldwide. Several risk factors for the development of a PPH exist. Recently, hematologic factors such as platelet count as well as factor XIII activity have come into focus. Factor XIII plays a major role in cross-linking fibrinogen and fibrin and is thus important in securing clot stability and increasing fibrinolytic resistance of the clot. Hence, it has been hypothesized that changes in FXIII might impact postpartum blood loss significantly.

Previously, our group observed that prepartum factor XIII activity has a strong impact on postpartum blood loss. Every 1% increase in prepartum factor XIII activity was associated with a (significantly increased) odds ratio of 1.011 to keep the measured blood loss below a given cut-off. This effect was shown to hold true across every statistical model and any clinical subgroup [1]. Similarly, other researchers found prepartum factor XIII activity to be significantly lower in women with PPH than in those with a normal peripartum blood loss [2, 3].

The prospective study carried out by our group between October 2015 and November 2016 in 1300 women allowed us not only to determine factor XIII activity, but also factor XIII subunit A (factor XIII antigen), which was measured with an immunological assay. To detect factor XIII activity, various functional factor XIII assays can be used (i.e., amine incorporation or isopeptidase assays). These are difficult to automate. However, albeit first steps toward automation having been undertaken [4]. While chromogenic ammonia release assays can readily be automated, they require a specific wavelength to be monitored [5] that is not available on all analyzers. Factor XIII (subunit A) antigen, on the other hand, can be measured via ELISA or latex agglutination immunoassays. The latter can be automated on analyzers with standard wavelength monitoring and is thus easier to include in a full laboratory automation [6], resulting in requirement for fewer personnel, which is especially important during night shifts [7]. Full automation also allows for results to be available after a shorter period of time [8, 9]. Because factor XIII activity has been shown to correlate well with factor XIII antigen in cases of factor XIII deficiency [10], we wanted to assess whether these results could be reproduced in case of potential FXIII depletion due to peripartum blood loss in our real-life population of more than 1300 women. If so, this would possibly allow for F XIII antigen assay implementation in emergency use such as in the case of postpartum hemorrhage.

## Methods

### Study design and study oversight

This is a secondary analysis of a prospective single-center cohort study [1], which was carried out at the Department of Obstetrics, University Hospital Zurich, Switzerland, from October 2015 to November 2016 after institutional review board approval (KEK-ZH 2015–0011, ClinicalTrials.gov registration NCT02604602, *“PPH-1300 study”*). The University Hospital Zurich, CSL Behring Switzerland, the Center for Laboratory Medicine St. Gallen and a private donor (a former patient) provided the funding for the study. CSL Behring and the private donor, as nonacademic entities, had neither an influence on the design or the execution of the study; nor on the collection, management, analysis, and interpretation of data; nor the preparation, review, or approval of the manuscript; nor on the publication.

Research staff used patient data from the general and obstetrics-specific clinical information systems for prospective data collection. The analyses for fibrinogen (Clauss assay), factor II (one stage clotting assay), and factor XIII (A subunit) were performed according to the recommendations of the manufacturer (IL, Werfen) on an ACL top 750 analyzer (IL, Werfen). The chromogenic factor XIII activity assay (Berichrom) was performed on a BCS XP analyzer (Siemens Healthineers), also according to the recommendations of the manufacturer. All coagulation assays were performed at the Center for Laboratory Medicine, Hemostasis and Hemophilia Center, St. Gallen, Switzerland, only after the last patient had been enrolled, thus preventing a potential treatment bias.

### Participants

Included were women admitted to the labor ward before vaginal delivery or cesarean section if they were ≥ 18 years of age and their pregnancy was ≥ 22 weeks of gestation. All study participants signed an informed consent before enrollment. Women with known congenital disorders of hemostasis, on anticoagulant therapy, and women with preeclampsia or eclampsia were not eligible. Enrollment was consecutive.

The prepartum blood sample was taken in the 36 h preceding the onset of labor; labor being defined as regular contractions or rupture of membranes (whichever occurred first). The postpartum blood sample was taken 24–48 h after delivery. Blood loss measurement followed a strict protocol [11], which had previously been validated and published: The protocol stipulates a fresh disposable draw sheet being placed underneath the woman’s hips after the delivery of the baby, which is thereafter regularly examined for blood. This approach ensures that the liquid on the draw sheet is blood rather than remaining amniotic fluid. If bleeding continues, the sheet is weighed on an infant scale which is located in every delivery suite. If the net weight (soaked sheet weight minus dry sheet weight) exceeded 300 g with the placenta still in utero or exceeded 500 g (irrespective if the placenta was still in utero or delivered), a sheet ending in a plastic bag labeled with 100 ml steps is placed under the woman’s pelvis to collect blood and further monitor the bleeding [1].

### Statistical analysis

Baseline demographics, fetomaternal and perinatal characteristics as well as prepartum coagulation factor values were stratified by mode of delivery. The respective descriptive statistics can be found in the supplementary material.

In a first step, the association of immunologically measured FXIII (A subunit) antigen with FXIII activity in the prepartum setting was calculated (Spearman Rank correlation coefficient). Further, a Bland–Altman plot was drawn to display the concordance between FXIII activity and FXIII antigen, and a Passing–Bablok regression was calculated to analyze systematic or proportional differences between the two values.

In a second step, the conditional distribution of MBL (measured blood loss) in relation to prepartum platelet count (G/L), hemoglobin (g/L), fibrinogen (g/L), FII (%), and FXIII antigen (%) was estimated by continuous outcome logistic regression [12, 13].

All possible binary logistic regression models for all MBL volumes were estimated in this model, hence allowing for applying this model to any blood loss cut-off point (i.e., the regression coefficients were treated as constants). This statistical approach and its validity are explained in detail in the original paper and its openly available supplements including codes and raw data [1]. In summary, the depicted odds ratio is calculated by regression coefficients and the model assesses the influence of a one-unit increase in each of the evaluated prepartum blood parameters on any given blood loss. These calculated odds ratios thus show the effect of a one-unit increase in the respective prepartum parameter on the probability to stay below any chosen MBL. Additionally, a binary logistic regression model using the conventional definition of PPH in vaginal deliveries as a cut-off, i.e., MBL of 500 ml, was used to compare the binary with the continuous logistic regression model.

## Results

**Fig. 1:**
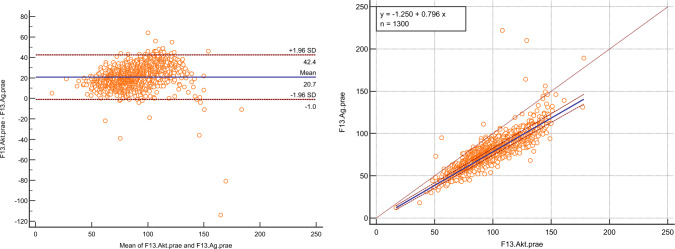
**A** Bland–Altman plot of FXIII activity and subunit A antigen. **B** Passing–Bablok regression of FXIII activity and antigen

Prepartum FXIII activity significantly correlated with prepartum FXIII antigen as shown by Spearman rank correlation (coefficient 0.89, *p* < 0.001). Bland–Altman testing revealed that FXIII activity was systematically measured higher than FXIII antigen (mean difference 20.7, 95% confidence interval − 1.0–42.4), Fig. 1A. Passing–Bablok regression confirmed the systematic difference between FXIII activity and FXIII antigen ([FXIII antigen] = − 1.250 + 0.796*[FXIII activity]), Fig. 1B. Both methods revealed that FXIII antigen values are roughly 20% lower than FXIII activity values.

Similarly to FXIII activity, prepartum FXIII (A subunit) antigen was significantly associated with measured blood loss: every one-unit increase in prepartum FXIII antigen was associated with an odds ratio of 1.009 (95% confidence interval, 1.005–1.014 *p* < 0.001) for postpartum blood loss to remain below any given volume (continuous outcome logistic regression model). Stratification of delivery mode yielded similar results with the effect size that had been observed in the continuous outcome logistic regression model, being significant for vaginal deliveries (OR 1.008, 95% CI 1.002–1.015, *p* = 0.016), and showing a trend, albeit not significant, for cesarean sections (OR 1.006, 95% CI 1.000–1.013, *p* = 0.06). Using the conventional cut-off for the definition of PPH in vaginal deliveries at MBL 500 ml, the respective odds ratio for F XIII antigen was 1.007 (95% CI 1.002–1.013, *p* = 0.01). The results and effect size estimation of the association of prepartum FXIII antigen with postpartum blood loss were thus comparable to the effect size of prepartum FXIII activity on postpartum blood loss in the original study.

**Fig. 2:**
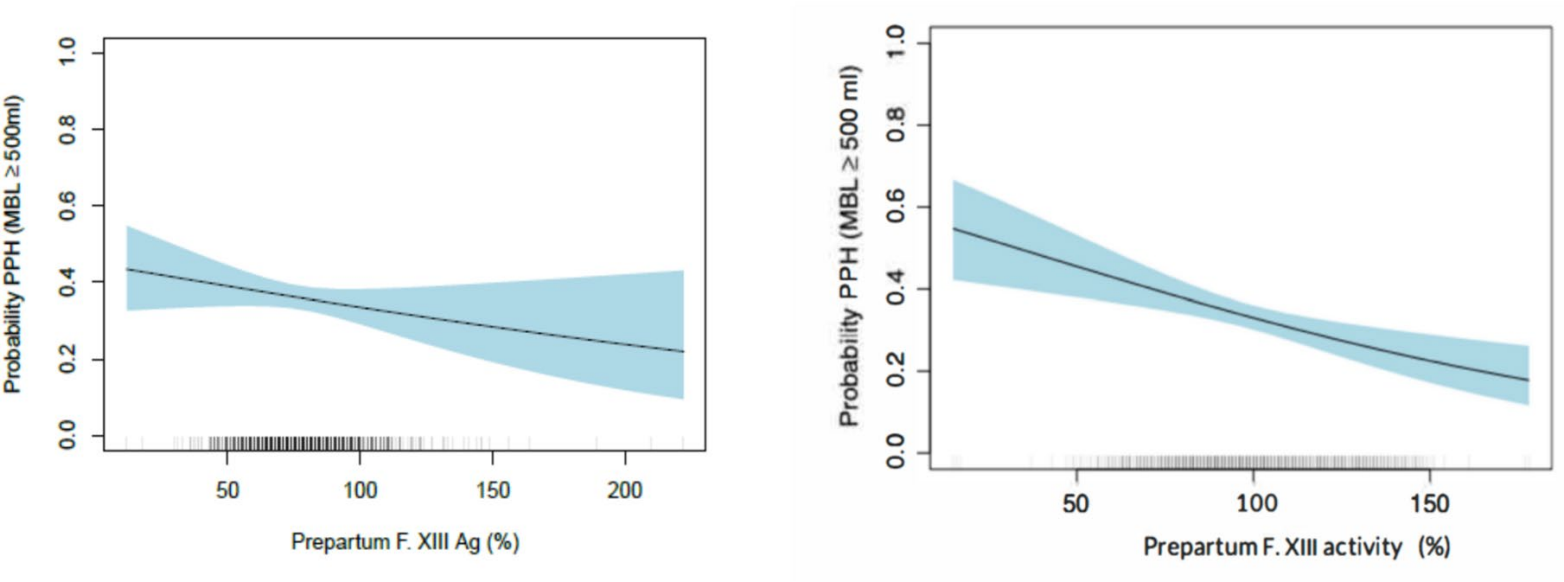
Prevalence of PPH (defined as MBL ≥ 500 ml) as a function of prepartum F. XIII Ag (left) and prepartum F. XIII activity (right) for a hypothetical subject with prepartum hemoglobin 127 g/l, prepartum F I 4.5 g/l, and prepartum F. II 128%. The right curve is taken from the original paper [1]. The blue shaded area represents a 95% confidence interval. Rungs on the horizontal axis represent the measured prepartum FXIII Ag and activity values, respectively; the darker a rung, the higher the number of patients with this measured FXIII Ag or activity value

After confirming the association between FXIII Ag and postpartum blood loss, a calculation to examine the overall prevalence of postpartum hemorrhage (defined as MBL ≥ 500 ml) relative to prepartum FXIII Ag levels was conducted, mirroring the approach taken with FXIII activity in Haslinger et al. 2020 [1] (Fig. 2).

## Discussion

In the last couple of years, the research spotlight has turned onto the potential clinical relevance of acquired FXIII deficiency as acquired loss of FXIII has been associated with clinically relevant consequences (such as the increased intraoperative use of blood products [10]) occurring more frequently than expected by random chance.

In the largest prospective observational study on postpartum hemorrhage to date, FXIII was shown to also have a significant effect on blood loss during delivery in the pregnant population, indicating that an increase in peripartum FXIII might lead to a reduction in postpartum blood loss [1]. Further prospective randomized intervention studies will have to be carried out to verify this observation, but in the meantime, the case for enzyme replacement in the form of FXIII has been strengthened.

However, FXIII (activity) assays are not available in every laboratory. As in the current moment, FXIII antigen (subunit A) levels can be more easily determined in an automated manner than FXIII activity, it seems worthwhile to evaluate the validity of FXIII antigen measurements in a clinical setting. In this secondary analysis of a prospective study of 1,300 parturient women, we could show that prepartum factor XIII activity strongly correlated with factor XIII antigen (subunit A) (rho = 0.89, *p* < 0.001). Although FXIII antigen showed a higher variance than FXIII activity with more outliers, the linear correlation is high.

As for the comparison of the two assays, the values measured for FXIII antigen were approximately 20% points lower than the measured FXIII activity as seen in the Bland–Altman plot. Such a picture has been observed previously in a comparative analysis between the same activity and a different antigen assay [10]; however, in this publication, the difference between the assays was only roughly 10%. Since “merely” 138 samples were compared, while our analysis comprised 1300 samples, the 10% discrepancy between the samples measured in the abovementioned publication and our data could be explained by the large difference in sample size. The Passing–Bablok regression analysis confirmed the difference of roughly 20% as displayed by the calculated formula (antigen = 0.80*activity–1.25). As the normal range of FXIII activity lies between 70 and 130% (− 140%, depending on the laboratory), the normal range of FXIII antigen values in a clinical setting would thus lie approximately between 50 and 110%.

Our results indicate that every one-unit (%) increase in prepartum FXIII antigen is associated with an odds ratio of 1.009 to remain below any volume of MBL and an odds ratio of 1.007 not to suffer from PPH (MBL ≥ 500 ml). Higher values of prepartum FXIII antigen are associated with a lower MBL. This mirrors the trend in the original paper, but the signal of FXIII antigen is not as strong as that of FXIII activity (OR 1.009 vs OR 1.011) [1]. When stratified for delivery mode, this effect was observed to be significant for vaginal deliveries and showed a trend for cesarean sections as well.

There are several limitations of our research. Even though our sample comprised 1300 women, the number of patients with a high measured blood loss during birth was low due to a low risk sample where women were consecutively enrolled in the trial: in the case of our sample, 191 patients with a vaginal delivery (spontaneous delivery/vacuum extraction) experienced a PPH (14.6%), but only 22 women had a MBL > 1500 ml (6 cesarean sections, 15 spontaneous deliveries and 1 vacuum extraction).

Also, at the time of the study, patients with a cesarean section still routinely received colloids. About 44% of patients in our sample received colloids (many without a PPH), a practice that has been completely discontinued in the past couple of years. Whether this has an effect on measured blood loss due to changes in coagulability has been discussed controversially in the literature [14]. However, the correlation of prepartum FXIII activity with FIII antigen could not have been influenced by the applied volume management.

Conversely, tranexamic acid was not yet administered routinely in all cases of a PPH, a practice that has also changed since the accrual period of our study as the results of the WOMAN trial have been implemented into clinical routine [15]. However, in this study, the emphasis was put on determining A) that FXIII activity and FXIII antigen correlate well, giving institutions that would like to assess FXIII activity but can only measure FXIII antigen a valid basis to do so, and B) that FXIII antigen showed a biological signal for the occurrence of PPH (at least in vaginal deliveries). The correlation coefficient of rho 0.89 and 0.902 demonstrates that FXIII activity significantly correlated with FXIII antigen both pre- and postpartum, respectively, and thus we argue that the determination of FXIII antigen might help us in an emergency setting. We can, however, not claim that the activity of prepartum FXIII can predict blood loss in general or that the two values have an equal precision to predict major blood losses.

Nonetheless, in emergency situations such as postpartum hemorrhage, to determine the level of FXIII in a fast and reliable manner seems useful. As it is likely that FXIII antigen measurements are easier to perform by a wide range of laboratories, measuring FXIII antigen rather than activity might increase the accessibility to FXIII determination in an emergency clinical setting. This large-scale real-world study involving more than 1300 parturient women shows that the results of FXIII antigen measurement are comparable, albeit not equal, to the results of FXIII activity measurement. The trend observed in the association of prepartum FXIII antigen values with postpartum blood loss mirrors that observed in the “*PPH1300-study”* analyzing the effect of prepartum FXIII activity on postpartum blood loss. FXIII antigen measurements could thus possibly be utilized in the emergency setting of a PPH when FXIII activity measurements are not available. Further studies looking at the effect of FXIII replenishment on MBL are ongoing and the results are awaited with anticipation.

## Supplementary Information

Below is the link to the electronic supplementary material.

Supplementary file1 (DOCX 17 KB)

## Data Availability

The datasets generated during and/or analysed during the current study are not publicly available due to the legal obligation to protect sensitive health care data of patients in Switzerland, but are available from the corresponding author on reasonable request.
