# Supplementary material for: Correlation of factor XIII subunit A with factor XIII activity in a population of parturient women
Source: Arch Gynecol Obstet. 2024 Nov 4;311(3):585–90. doi: 10.1007/s00404-024-07799-2 (PMC11920330; doi:10.1007/s00404-024-07799-2)
Supplement: Supplementary file 1 — Supplementary file1 (DOCX 17 KB) [file 404_2024_7799_MOESM1_ESM.docx]

**Supplementary material**

**Table 1** Fetomaternal and perinatal characteristics, prepartum hemoglobin, prepartum factor XIII activity and antigen and other blood coagulation factors, stratified by mode of delivery.

| Variable | Vaginal delivery (N = 677) | Elective cesarean delivery (N = 409) | Unplanned cesarean delivery (N = 223) |
| --- | --- | --- | --- |
| Measured blood loss, mL | 350 (300–500) | 500 (400-600) | 500 (400–700) |
| Prepartum hemoglobin, g/L | 128.0 (121.0–135.0) | 124.0 (118.0-131.0) | 127.0 (120.0-134.0) |
| Prepartum fibrinogen, g/L | 4.5 (3.9–5.1) | 4.3 (3.9-4.8) | 4.5 (3.9-5.2) |
| Prepartum factor II activity, % | 128.0 (118.0–140.0) | 128.0 (115.0-138.0) | 128.0 (115.0-140.0) |
| Prepartum factor XIII activity, % | 98.5 (86.0–117.8) | 93.0 (82.0-107.0) | 93.0 (80.2-111.0) |
| Prepartum factor XIII antigen, % | 78.0 (67.0–91.0) | 74 (65.0-84.0) | 74.0 (63.0-88.0) |
| Spontaneous delivery | 566 | - | - |
| Vacuum delivery | 111 | - | - |
| Unplanned cesarean delivery (non-urgent) | - | - | 210 |
| Unplanned cesarean delivery (emergency) | - | - | 13 |
| Gestational age, days | 280 (273-285) | 267 (265-270) | 277 (268-284) |
| Maternal age, years | 32 (29-35) | 34 (30-37) | 33 (30-36) |
| Multiparity | 310 (45.8%) | 254 (62.1%) | 70 (31.4%) |
| Body mass index, kg/m^2^ | 23.2 (20.5-26.8) | 25.1 (21.7-28.8) | 23.4 (21.0-26.4) |
| Duration of second stage labor, minutes | 51 (18-121) | - | 173 (119-207) |
| Multiple fetus pregnancy | 6 (0.9%) | 32 (7.8%) | 13 (5.8%) |
| Induction of labor | 263 (38.8%) | 4 (1.0%) | 85 (38.1%) |
| Induction of labor >48 hours | 22 (3.2%) | 1 (0.2%) | 18 (8.1%) |
| Chorioamnionitis | 1 (0.1%) | 0 | 9 (4.0%) |
| Neonatal weight, g | 3370 (3090-3650) | 3200 (2890-3510) | 3340 (2945-3690) |
| Uterine rupture | 0 | 0 | 3 (1.3%) |
| Uterine atony | 42 (6.2%) | 7 (1.7%) | 4 (1.8%) |
| Retained placenta | 24 (3.5%) | 0 | 0 |
| Retained placental tissue | 26 (3.8%) | 1 (0.2%) | 0 |
| Morbidly adherent placenta | 1 (0.1%) | 3 (0.7%) | 1 (0.4%) |
| Placenta previa | 0 | 9 (2.2%) | 4 (1.8%) |
| Bleeding from laceration | 49 (7.2%) | 0 | 0 |
| Placental abruption | 2 (0.3%) | 2 (4.9%) | 6 (2.7%) |

Data are median (interquartile range) or n (%)
